# Supplementary figures and images for: Beyond dissolution: Xerostomia rinses affect composition and structure of biomimetic dental mineral in vitro
Source: PLoS One. 2021 Apr 26;16(4):e0250822. doi: 10.1371/journal.pone.0250822 (PMC8075190; doi:10.1371/journal.pone.0250822)

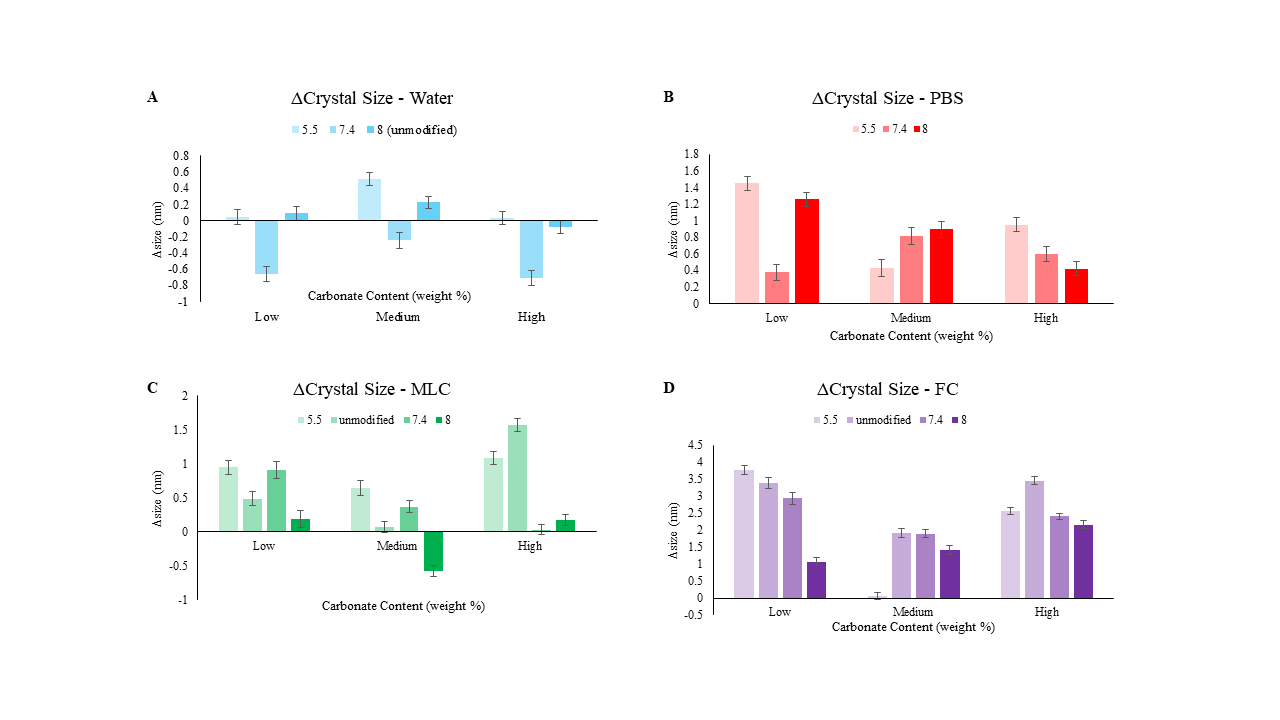

Supplement: S1 Fig — CAP has variations in crystal size after exposure to water, suggesting that the internal structure does not change (A). The crystallite size of CAP increased in PBS, MLC, and FC, indicating that CAP is becoming more crystalline after exposure (B-D). Error bars account for standard deviations related to the slope derived from the Halder-Wagner equation. (TIF) [file pone.0250822.s001.tif]

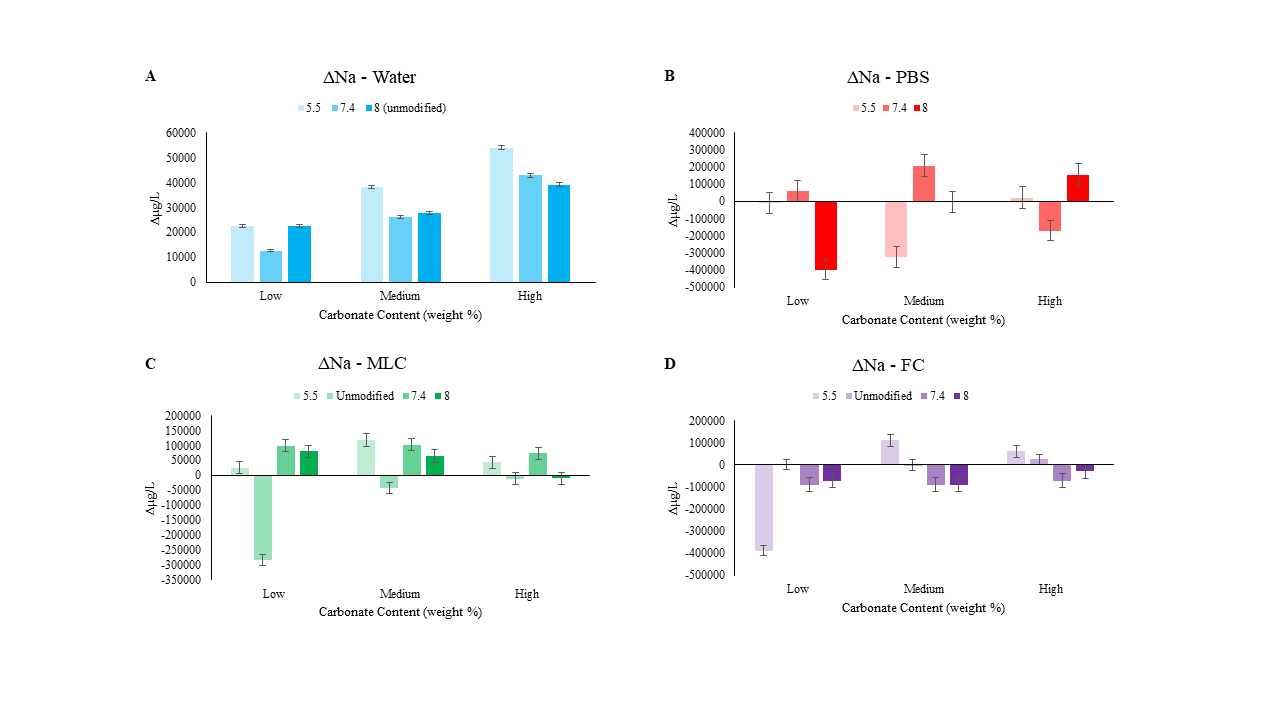

Supplement: S2 Fig — An increase in sodium in water may be due to a low sodium content in the pre-exposure water and indicates that CAP is releasing sodium during exposure (A). The amount of sodium in solution varied in PBS, MLC, and FC irrespective of pHi and powder carbonate content (B-D). (TIF) [file pone.0250822.s002.tif]

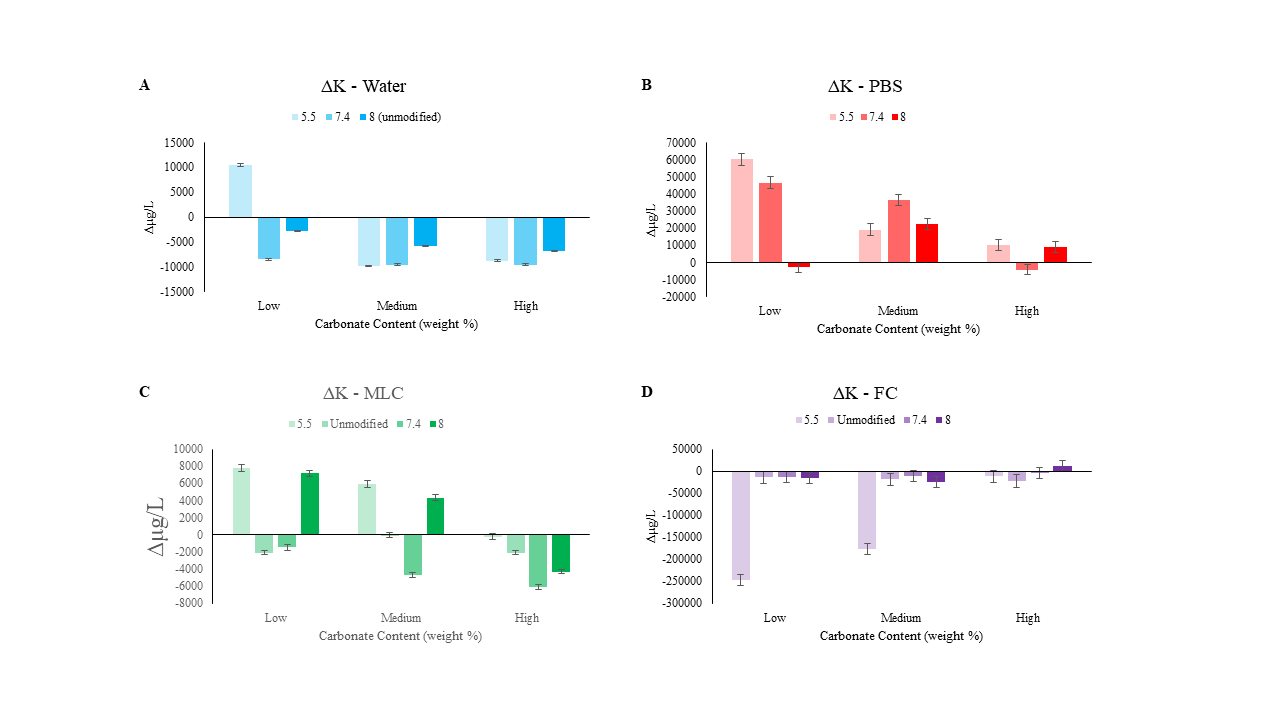

Supplement: S3 Fig — Potassium generally increased in PBS, revealing that apatite is releasing potassium (B). The opposite is shown for FC, where the decrease in potassium suggests that CAP is up taking into the structure during recrystallization to account for charge balance of the crystals (D). MLC and water were variable, implying that potassium has little effect in these solutions (A, C). (TIF) [file pone.0250822.s003.tif]
