# Supplementary material for: Beyond dissolution: Xerostomia rinses affect composition and structure of biomimetic dental mineral in vitro
Source: PLoS One. 2021 Apr 26;16(4):e0250822. doi: 10.1371/journal.pone.0250822 (PMC8075190; doi:10.1371/journal.pone.0250822)
Supplement: S1 Table — (DOCX) [file pone.0250822.s004.docx]

**S1 Table. List of ingredients for Biotene and ACT.**

| **Biotene:**  Purified Water,  Glycerin,  Xylitol,  Sorbitol, Propylene Glycol,  Poloxamer 407,  Sodium Benzoate,  Hydroxyethyl Cellulose, Methylparaben,  Propylparaben,  Flavor, Sodium Phosphate,  Disodium Phosphate | **ACT:**  Sodium fluoride 0.02%  Water,  Glycerin,  Sorbitol,  Xylitol,  Poloxamer 407,  Betaine,  Propylene glycol,  Flavors,  Bisabolol,  Carnosine,  PEG-14M,  PEG-160M,  Zingiber officinale (ginger) root extract,  Angelica polymorpha sinensis root extract,  Lonicera japonica (honeysuckle) flower extract,  Pueraria lobata root extract,  Sodium benzoate,  Potassium sorbate,  Sodium phosphate,  Disodium phosphate,  Polysorbate 20,  Lactic acid,  Calcium disodium EDTA,  Cetylpyridinium chloride,  Sucralose,  Green 3,  Yellow 10 (309-043) |
| --- | --- |

The list of ingredients in the order as shown on the bottles. Highlighted in yellow are the ingredients that are common between the dry mouth rinses.
