# Supplementary material for: Beyond dissolution: Xerostomia rinses affect composition and structure of biomimetic dental mineral in vitro
Source: PLoS One. 2021 Apr 26;16(4):e0250822. doi: 10.1371/journal.pone.0250822 (PMC8075190; doi:10.1371/journal.pone.0250822)
Supplement: Attachment — Submitted filename: 210327_ResponsetoReviewers.docx [file pone.0250822.s005.docx]

Reviewer #1:

**Comment 1: The objective of this study was to elucidate the effects of xerostomia rinse composition on dentin like biomimetic apatites. The authors applied a multi-technique approach to understanding the effects of four different solutions containing: aqueous bases, high phosphate levels, mucin-like molecules, and fluoride. The combination of mass loss, pH, Raman spectroscopy, Inductively Coupled Plasma Optical Emission Spectrometry (ICP-OES), and X-ray diffraction (XRD) measurements allowed us to provide a comprehensive view of how these solutions modify dental tissues at a compositional and structural level.**

Thank you for the supportive comments. We appreciate your understanding of the value of examining the effect of clinically relevant xerostomia treatments on the behavior of biomimetic apatites for dental health.

**Comment 1: It is an in vitro study and must appear in the title**

We agree with the reviewer and have changed the title to read “Beyond dissolution: xerostomia rinses affect composition and structure of biomimetic dental mineral in vitro”.

**Comment 2: The study design is not clear to readers. It is very narrative in some sections and should be synthesized.**

We have made extensive revisions to the text in both organization and format to clarify the study design. The methods and discussion sections were rewritten to eliminate unnecessary verbiage and detail to reduce the prior narrative nature. Please see all highlighted changes in the text.

**Comment 3: The authors use biotene and APC substances with different and many different active compounds are difficult to assess. Purified Water,Glycerin,Xylitol,Sorbitol, Propylene Glycol,Poloxamer ,sodium Benzoate…Hydroxyethyl Cellulose,Zingiber officinale (ginger) root extract,Angelica polymorpha sinensis root extract,Lactic acid,……………**

Thank you for your comment regarding the different ingredients. We agree with the reviewer that the additional components in the Biotene and ACT rinses complicate the exact mechanisms of dissolution/recrystallization of CHA in these two solutions. With that in mind, we wanted the solutions to be clinically relevant. This has been emphasized in the introduction on page 2-3, which now reads:

“As a result, the current available research fails to truly elucidate the effect of oral rinse composition on dissolution and ignores the effect of these treatments on the remaining tooth mineral composition and structure. **In addition, basic science studies examining the effects of specific ions/molecules on mineral content fail to provide clear clinically-relevant relationships about current treatment options**.

In this study, we seek to elucidate the effect of clinically-relevant oral rinse composition on the dissolution, composition and structure of biomimetic dental apatites. We applied a multi-technique approach to understand the effects of four different clinically-relevant solutions containing: aqueous bases, high phosphate levels, mucin-like molecules, and fluoride.”

This is also emphasized in the methods sections on page 3-4 under the “Preparation of solutions” section:

“To determine the effects of oral rinse composition on biomimetic dental mineral, four solutions were selected: (1) Tap water (aqueous base) (2) 1X phosphate buffered saline (PBS - aqueous base with additional phosphate), (3) Biotène Dry Mouth Oral Rinse® (mucin like containing - MLC), and (4) ACT® Anticavity Total Fluoride Mouthwash (fluoride containing - FC). The tap water was sourced from the Farmington, CT water supply. PBS was diluted from 10X PBS (Fisher Scientific, Waltham, MA) in MilliQ water. Biotène and ACT oral rinses were purchased from a local pharmacy. Biotène and ACT represent some of the most prescribed oral rinses for xerostomia. Although they have complex compositions, these 2 solutions were selected for the presence of mucin-like molecules such as hydroxyethyl cellulose in Biotène and the presence of high levels of fluoride in ACT (Table S1), **making them unique and clinically-relevant solutions**.”

We chose not to address the role of the many ingredients in Biotene and ACT that may play in dissolution/reprecipitation. This was in part due to low concentrations of the ingredients or previous literature data suggesting a lack of any effect. While we do not know the precise concentration of certain ingredients in the solutions due to patent rights, we do know the order of ingredients on the label, which are in the order of greatest concentration to lowest concentration^1^. The first 8 ingredients in Biotene are largely responsible for solution mouthfeel, taste, and solubility (Table S1). For example, poloxamer 407 is a surfactant^2^, sodium benzoate is a preservative^2^, xylitol/sorbitol are sugar-substitutes^3^, and the viscosity of glycerin serves as an emulsifier and improves mouth feel^4,5^. There is limited or no information relating these compounds as well as others like parabens, root and flower extracts, and food coloring, to tooth dissolution and recrystallization. However, we do have some information about how some interact with tooth structures and dental health.

The possible anti-cariogenic properties of xylitol and sorbitol have been extensively studied. It is suggested that these properties likely have to do with their interactions with oral bacteria; however, the literature results are mixed^3,6–12^. There is some evidence that xylitol may act as a Ca^2+^ transporter and affect mineralization^11^; however, the American Dental Association (ADA) currently classifies the xylitol data as insufficient^13^. Lactic acid in ACT might also be expected to have a demineralizing effect^14^. This might be in part responsible for the lower unmodified pH of the solution, although its position in the list of ingredients suggests a low concentration. Any acidification would be measured from the pH evaluations in this study. Food colorings, known as extrinsic chemogens, have been shown to accumulate in the surface deposits of teeth, specifically in the acquired pellicle layer^15,16^. We found that none of our powders exhibited a color change after exposure to the oral rinse solutions, likely due to a lack of pellicle layer in our in vitro powder model. While many of the ingredients in Biotene overlap with those in ACT, a notable exception is the addition of hydroxyethyl cellulose in Biotene. This mucin-like molecule has been implicated in the dissolution/remineralization mechanisms in previous studies as stated on page 17-18, making this a probable component of changing apatite properties. The role of hydroxyethyl cellulose in our studies remains unclear. Comparisons of Raman spectra of the apatites before and after exposure, as well as spectra from Biotene and ACT showed that there was generally no binding of additional components onto the apatites after rinsing. In a few cases however, Biotene exposed samples did exhibit Biotene associated peaks that match the spectra of hydroxyethyl cellulose^17^ after a single rinse with Millipore water (Figure 1). However, the Raman spectra of CHA showed no peaks from Biotene after a second rinse. The powders did not exhibit variations in crystal lattice suggesting the incorporation of large molecules, as shown by their XRD patterns (Figure 2). This suggests that there might be weak bonding between the hydroxyethyl cellulose and the apatite surfaces. This has led us to suggest on page 17-18 that interactions between this mucin-like molecule and the apatite in solution may serve as a protective coating during dissolution/recrystallization processes.

**Figure 1.** Raman spectra of Biotene solution (blue), 6% CHA after Biotene exposure (purple), 6% CHA in Biotene re-rinsed with water (green), 6% CHA after ACT exposure and water rinse (orange). Dashed vertical lines indicate the respective peaks of apatite.

In ACT, the first ingredient listed is 0.02% NaF (Table S1). Fluoride has long been known to substitute into apatite lattices to form fluorapatite, which exhibits decreased solubility and increased resistance to caries. We therefore propose that the biggest influencer of apatite dissolution/recrystallization process in ACT is the fluoride. Peaks shifts, decreases in peak width in our XRD data, as well as the significant carbonate loss from our Raman data indicate that there is a transition from the carbonated apatite to the more crystalline fluorapatite (Figure 2). This supports our theory that fluoride acts as a primary contributor to apatite dissolution/recrystallization behaviors.

**Figure 2.** XRD patterns of 6% CHA powders in ACT (top) and Biotene (bottom) post-exposure.

Based on the current available literature and our goal to elucidate the effects of the rinses as a whole on dissolution of dental mineral, we suggest that fluoride and MLCs are likely the most relevant components in the dissolution/recrystallization mechanisms of CHA in ACT and Biotene, respectively. This is concisely addressed in the discussion under “Experimental limitations” on page 19, which reads: “While there are other ingredients in the FC and MLC rinses that could be affecting the dissolution seen in these experiments, the interactions between mucin-like molecules and fluoride on teeth have been previously shown to have the greatest effect on apatite mineral; therefore, we have chosen to focus on these factors instead of the many additional ingredients. We believe that this study plays a crucial role in beginning to elucidate properties of these components.”

Although it would be of great interest to examine the effects of each individual component, it is beyond the scope of this paper, whose goal was to elucidate how the rinses as a whole elicit clinically relevant dissolution responses on dental mineral.

**Table S1. List of ingredients for Biotene and ACT.**

| **Biotene:**  Purified Water,  Glycerin,  Xylitol,  Sorbitol, Propylene Glycol,  Poloxamer 407,  Sodium Benzoate,  Hydroxyethyl Cellulose, Methylparaben,  Propylparaben,  Flavor, Sodium Phosphate,  Disodium Phosphate | **ACT:**  Sodium fluoride 0.02%  Water,  Glycerin,  Sorbitol,  Xylitol,  Poloxamer 407,  Betaine,  Propylene glycol,  Flavors,  Bisabolol,  Carnosine,  PEG-14M,  PEG-160M,  Zingiber officinale (ginger) root extract,  Angelica polymorpha sinensis root extract,  Lonicera japonica (honeysuckle) flower extract,  Pueraria lobata root extract,  Sodium benzoate,  Potassium sorbate,  Sodium phosphate,  Disodium phosphate,  Polysorbate 20,  Lactic acid,  Calcium disodium EDTA,  Cetylpyridinium chloride,  Sucralose,  Green 3,  Yellow 10 (309-043) |
| --- | --- |

**Comment 4: Another limitation is that A-type CO3 substitutions are not accounted for in this study.**

We agree that it is important to understand how the carbonate is distributed across the crystals during dissolution and recrystallization, as changes in the amount and location of carbonate substitutions can significantly affect the structure, morphology, solubility, mechanics, and buffering ability of apatites^18–20^. Historically, B-type substitutions have been found to be the most prominent type of carbonate substitutions in biological apatites^21^ due to its lower energy barrier for formation^18^. Conversely, A-type carbonate is more present in geologic and synthetic apatites exposed to high temperature and pressure conditions^22–24^. Since we create our apatites at 60℃ at local atmospheric pressure, this suggests that A-type carbonate is not the primary substitution involved in our teeth. However, there has been growing debate about the validity of methods like Fourier Transform Infrared Spectroscopy (FTIR), and Thermogravimetric Analysis (TGA) to identify differences in A- and B-type apatites.

Overlapping of FTIR peaks associated with B-, A- and labile (A2) carbonates in apatites can make it difficult to deconvolute individual peaks^25^ (Figure 3). Our unpublished work with Dr. Claude Yoder examining bone apatites with FTIR has shown that relative amounts of A- vs B- type CO3 can vary depending on whether we deconvolve the v_2_ CO3 or the v_3_ CO3 peaks of the apatite. However, our XRD data, in agreement with other studies on biological apatites, indicate that our apatite models are more B-type apatites^26,27^.

**Figure 3.** ATR-FTIR spectra of the v2 CO3 peaks of the mineral in ox bone exposed to various aqueous solutions^28^. Modified from Aufort, J., et al., Palaeogeogr. Palaeoclimatol. Palaeoecol., 526:80-95, 2019.

We are currently expanding our research interests to include the roles of A- and B- type carbonate on apatite dissolution/recrystallization and buffering using FTIR. We are also applying Nuclear Magnetic Resonance (NMR) to further differentiate A-type carbonate levels. Although extremely interesting, these additional techniques are beyond the scope of this work. We have identified the lack of information about carbonate location in the paper on page 19 which reads:

“Another limitation is that A-type CO_3_^2-^ substitutions are not accounted for in this study. Although small quantities of A-type CO_3_^2-^ are present in bones and teeth, B-type CO_3_^2-^ substitutions are predominantly found in these tissues [25; 53; 54], similar to our synthesized apatites. The changes in the lattice spacing from XRD indeed indicates that the bulk of the substitutions are B-type CO_3_^2-^ in our system and not A-type CO_3_^2-^. However, Raman is unable to distinguish between the two substitution types making it difficult to determine the role of A-type CO_3_^2-^ in this study.”

**Comment 5: Graphics have poor quality**

Thank you for bringing this to our attention. Our original figures met the resolution requirements of the journal; however, they appeared blurry during the journal’s upload process. We have further improved the resolution for this submission; however, once again there seem to be issues with the upload on the website. We have brought this to the editors attention but we recommend that you click on the link for each figure page which seems to provide access to our high resolution images.

**Comment 6: I should provide some figure of the raman with the different components**

We appreciate this comment. We see the added value of including the Raman spectra of the apatites in the different solutions to our figures. We included the spectra of 6% carbonated apatite in all 4 solutions at pH 5.5 to Figure 5. The shift of the v_1_ PO4 peak at 960 cm^-1^ in the ACT sample as well as the narrowing of the peaks will further validate our findings regarding the fluoride contribution to CHA dissolution/recrystallization to a more fluorinated apatite.

This has been referred to in the section titled “Fluoride in FC solution promoted reprecipitation of fluorapatite” on page 18, which reads:

“However, in this case the reprecipitated crystal is not a low-carbonate apatite, but fluorapatite, as suggested by the peak shift of the v_1_ PO4 in the Raman spectra (Fig. 5E). In addition, comparison of XRD spectra with standards from the PDF 2001 library showed that the post-exposure powders exhibit a greater resemblance to fluorapatite than carbonated apatite. The decrease in both d_C_ and d_A_ supports this removal of CO_3_^2-^ and formation of fluorapatite since fluorapatite exhibits a smaller d_A_ than carbonated apatites [49]. This phase change decreased ε_RMS_ and increased crystal size, as expected for the more crystalline fluorapatite. This reprecipitation also resulted in a decrease in solution phosphorus levels as it was likely taken up to form fluorapatite crystals (Fig. 8). Since fluorapatite has a lower density than hydroxyapatite, this may explain the larger mass loss with FC rinse exposure. Overall, this highlights the need to look beyond dissolution alone to understand how dental mineral will be affected by solution exposure.”

REFERENCES:

1.	FDA. Why Are Food and Color Ingredients Added to Food? *https://www.fda.gov/downloads/Food/FoodIngredientsPackaging/ucm094249.pdf* 1–13 (2010).

2.	Chemical, S. Mouthwash Ingredients. spec-001_ds_mouthwash_v3_new.pdf.

3.	Maguire, A. & Rugg-Gunn, A. J. *Xylitol and caries prevention-is it a magic bullet?* *BRITISH DENTAL JOURNAL VOLUME* vol. 194 (2003).

4.	Becker, L. C. *et al.* Expert Panel Safety Assessments Safety Assessment of Glycerin as Used in Cosmetics. doi:10.1177/1091581819883820.

5.	Padmawar, A., Bhadoriya, U. & Padmawar, A. R. *GLYCOL AND GLYCERIN: PIVOTAL ROLE IN HERBAL INDUSTRY AS SOLVENT/CO-SOLVENT*. www.wjpmr.com (2018).

6.	Riley, P., Moore, D., Ahmed, F., Sharif, M. O. & Worthington, H. V. Xylitol and Caries prevention. *Cochrane Database of Systematic Reviews* vol. 2015 (2015).

7.	Gonçalves, N. C. L. A. V *et al.* Effect of xylitol:sorbitol on fluoride enamel demineralization reduction in situ. doi:10.1016/j.jdent.2005.12.008.

8.	Cardoso, C. A. B. *et al.* Effect of xylitol varnishes on remineralization of artificial enamel caries lesions in vitro. doi:10.1016/j.jdent.2014.08.009.

9.	Chunmuang, S., Jitpukdeebodintra, S., Chuenarrom, C. & Benjakul, P. Effect of xylitol and fluoride on enamel erosion in vitro. *J. Oral Sci.* **49**, 293–297 (2007).

10.	Rochel, I. D. *et al.* Effect of experimental xylitol and fluoride-containing dentifrices on enamel erosion with or without abrasion in vitro. *J. Oral Sci.* **53**, 163–168 (2011).

11.	Miake, Y., Saeki, Y., Takahashi, M. & Yanagisawa, T. *Remineralization effects of xylitol on demineralized enamel*. *Journal of Electron Microscopy* vol. 52 https://academic.oup.com/jmicro/article/52/5/471/1044146 (2003).

12.	Tuncer, D., Onen, A. & Yazici, A. R. Effect of chewing gums with xylitol, sorbitol and xylitol-sorbitol on the remineralization and hardness of initial enamel lesions in situ. *Dent. Res. J. (Isfahan).* **11**, 537–43 (2014).

13.	New research shows clinical evidence unclear on effects of xylitol products preventing dental caries. https://www.ada.org/en/publications/ada-news/2015-archive/march/new-research-shows-clinical-evidence-unclear-on-effects-of-xylitol-products-preventing-dental-carie (2015).

14.	Harper, R. A. *et al.* Acid-induced demineralisation of human enamel as a function of time and pH observed using X-ray and polarised light imaging. *Acta Biomater.* **120**, 240–248 (2020).

15.	Addy, M. & Moran, J. *MECHANISMS OF STAIN FORMATION ON TEETH, IN PARTICULAR ASSOCIATED WITH METAL IONS AND ANTISEPTICS*. *Adv Dent Res* vol. 9 (1995).

16.	Watts, A. & Addy, M. Tooth discolouration and staining: A review of the literature. *Br. Dent. J.* **190**, 309–316 (2001).

17.	John Wiley & Sons, I. S. SpectraBase Compound. ID=J4sEjBlmZV SpectraBase.

18.	Madupalli, H., Pavan, B. & Tecklenburg, M. M. J. Carbonate substitution in the mineral component of bone: Discriminating the structural changes, simultaneously imposed by carbonate in A and B sites of apatite. *J. Solid State Chem.* **255**, 27–35 (2017).

19.	Peccati, F., Bernocco, C., Ugliengo, P. & Corno, M. Properties and Reactivity toward Water of A Type Carbonated Apatite and Hydroxyapatite Surfaces. *J. Phys. Chem. C* **122**, 3934–3944 (2018).

20.	Yoder, C. H., Stepien, K. R. & Edner, T. M. A new model for the rationalization of the thermal behavior of carbonated apatites. *J. Therm. Anal. Calorim.* **140**, 2179–2184 (2020).

21.	Bigi, A. *et al.* Chemical and structural characterization of the mineral phase from cortical and trabecular bone. *J. Inorg. Biochem.* **68**, 45–51 (1997).

22.	Tacker, R. C. & Chris Tacker, R. Carbonate in igneous and metamorphic fluorapatite: Two type A and two type B substitutions. *Am. Mineral.* **93**, 168–176 (2008).

23.	Fleet, M. E. & Liu, X. Location of type B carbonate ion in type A-B carbonate apatite synthesized at high pressure. *J. Solid State Chem.* **177**, 3174–3182 (2004).

24.	Kubota, T., Nakamura, A., Toyoura, K. & Matsunaga, K. The effect of chemical potential on the thermodynamic stability of carbonate ions in hydroxyapatite. *Acta Biomater.* **10**, 3716–3722 (2014).

25.	Spizzirri, P. G., Cochrane, N. J., Prawer, S. & Reynolds, E. C. A Comparative Study of Carbonate Determination in Human Teeth Using Raman Spectroscopy. *Caries Res.* **46**, 353–360 (2012).

26.	Akkus, O., Adar, F. & Schaffler, M. B. Age-related changes in physicochemical properties of mineral crystals are related to impaired mechanical function of cortical bone. *Bone* **34**, 443–453 (2004).

27.	Deymier, A. C. *et al.* Protein-free formation of bone-like apatite: New insights into the key role of carbonation. *Biomaterials* **127**, 75–88 (2017).

28.	Aufort, J. *et al.* Atomic scale transformation of bone in controlled aqueous alteration experiments. *Palaeogeogr. Palaeoclimatol. Palaeoecol.* **526**, 80–95 (2019).
